# The association of attention deficit hyperactivity disorder with socioeconomic disadvantage: alternative explanations and evidence

**DOI:** 10.1111/jcpp.12170

**Published:** 2013-11-26

**Authors:** Ginny Russell, Tamsin Ford, Rachel Rosenberg, Susan Kelly

**Affiliations:** 1 ESRC Centre for Genomics in Society (Egenis) & Institute of Health Research, University of Exeter Medical SchoolExeter, UK; 2 Institute of Health Research, University of Exeter Medical SchoolExeter, UK; 3 Centre for Longitudinal Studies, Institute of EducationLondon, UK

**Keywords:** ADHD, child development, longitudinal studies, social class, sociocultural influence

## Abstract

**Background:**

Studies throughout Northern Europe, the United States and Australia have found an association between childhood attention deficit hyperactivity disorder (ADHD) and family socioeconomic disadvantage. We report further evidence for the association and review potential causal pathways that might explain the link.

**Method:**

Secondary analysis of a UK birth cohort (the Millennium Cohort Study, *N* = 19,519) was used to model the association of ADHD with socioeconomic disadvantage and assess evidence for several potential explanatory pathways. The case definition of ADHD was a parent-report of whether ADHD had been identified by a medical doctor or health professional when children were 7 years old.

**Results:**

ADHD was associated with a range of indicators of social and economic disadvantage including poverty, housing tenure, maternal education, income, lone parenthood and younger motherhood. There was no evidence to suggest childhood ADHD was a causal factor of socioeconomic disadvantage: income did not decrease for parents of children with ADHD compared to controls over the 7-year study period. No clinical bias towards labelling ADHD in low SES groups was detected. There was evidence to suggest that parent attachment/family conflict mediated the relationship between ADHD and SES.

**Conclusion:**

Although genetic and neurological determinants may be the primary predictors of difficulties with activity level and attention, aetiology appears to be influenced by socioeconomic situation.

## Introduction

Childhood attention deficit hyperactivity disorder (ADHD) has been reported to be more prevalent among socioeconomically disadvantaged groups in many regions of the developed world. Studies from the United States (Akinbami, Liu, Pastor, & Reuben, 2011; Froehlich et al., 2007; Pastor & Rueben, 2008; St Sauver et al., 2004), the United Kingdom (Ford, Collishaw, Meltzer, & Goodman, 2007) and Scandinavian countries (Bøe, Øverland, Lundervold, & Hysing, 2012; Hjern, Weitoft, & Lindblad, 2010; Paananen et al., 2012), as well as in Australia (Sciberras, Ukoumunne, & Efron, 2011) and Germany (Döpfner, Breuer, Wille, Erhart, & Ravens-Sieberer, 2008), have all found an association between increased childhood ADHD or behavioural symptoms of ADHD and socioeconomic disadvantage. A recent systematic review, although focused on treatments for ADHD, noted that both symptoms and diagnosis of ADHD are more common among those from a low socioeconomic status (SES) background (Charach et al., 2011).

**Figure 1:**
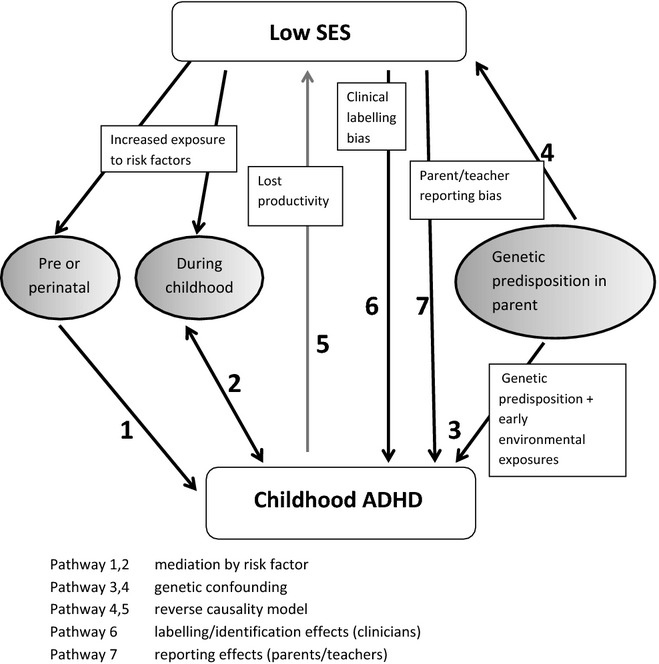
Simplified schematic illustration of the potential explanations for the association of ADHD with socioeconomic disadvantage.

ADHD is diagnosed when a child demonstrates inattentive, hyperactive and impulsive behaviours in multiple settings which cause functional impairment (American Psychiatric Association, 2013). Potential explanations for the association can be classified into two types. First ‘real’ effects: in lower socioeconomic groups children truly have higher symptom levels. Second, ‘labelling’ effects: greater awareness and access to health care in some groups or differential reporting about the same level of difficulties between groups (Boyle et al., 2011). Figure1 provides a schematic illustration of the causal pathways that may explain the link between childhood ADHD and low SES.

Proponents of health inequalities models have tended to position disease as an effect of socioeconomic disadvantage (Najman et al., 2004), often operating through differential exposure (Pathways 1 and 2, Figure1). In this pathway, higher rates of ADHD in groups with greater socioeconomic disadvantage are mediated through differential exposure. Such exposures could be perinatal, prenatal or occur during childhood. A systematic review of pre- and perinatal risk factors for ADHD only implicated exposure to tobacco smoke in utero as a suspected risk factor (Linnet et al., 2003). Several studies have shown association between smoking in pregnancy and increased risk of ADHD (Schothorst & Van Engeland, 1996; Thapar et al., 2003) although other research suggests genetic and socioeconomic confounders partially or entirely account for the effect (e.g. Lindblad & Hjern, 2010).

Exposures later in childhood have also been linked to ADHD phenotypes and socioeconomic disadvantage; for example, numerous studies have examined the link between parenting and ADHD, several describing unattached parenting or family conflict as risk factors (Deault, 2010; Johnston & Mash, 2001). Pathway 2, Figure1 illustrates influence of mediating factors in childhood, of which family conflict is the focus in our analysis, although there is evidence for various other risk factors during this stage. For example, a randomised double blind placebo-controlled trial found augmented food additives in the diet led to increased hyperactivity in children (McCann et al., 2007).

Pathways 3 and 4 (Figure1) illustrate genetic and neurological explanations of causality. An average estimate of heritability of ADHD was derived at 76%, from 20 twin studies (Faraone et al., 2005) although the effect of gene-environment interactions are hard to separate from purely genetic influences. Adoption studies suggest ADHD has a strong genetic component, but even these designs cannot discount the influence of prenatal environmental risks (Thapar, Cooper, Eyre, & Langley, 2013). No design to date has separated inheritance due to shared environmental influences from genetic influences convincingly. Specific genetic risks identified so far for ADHD tend to have small effect sizes or to be rare and often increase risk of many other types of psychopathology. Thapar et al. (2013) propose that the separation of genetic from environmental influences is a false dichotomy: ADHD is a multifactorial complex condition with many genes acting together to affect predisposition while environment acts on the genotype for the ADHD phenotype to present itself. Genetic predisposition in parents may lead to inherited predisposition in children with expression triggered by environmental factors (Pathway 3). ADHD symptoms in parents cause them difficulties in maintaining relationships and lead to poor socioeconomic outcomes (Pathway 4), as seen in recent studies of outcome at adulthood (e.g. Galéra et al., 2013). In the genetic confounding scenario, parents of children with ADHD have a genetic predisposition to hyperactive and inattentive behaviours themselves, and are therefore more likely to (a) pass on such a predisposition to their children and (b) suffer socioeconomic disadvantage described above.

Pathways 4 and 5 (Figure1) illustrate pathways that conceptualise ADHD as in itself a cause of low SES (reverse causality). A meta-analysis by Doshi et al. (2012) estimated national productivity losses due to family members with children who had ADHD at $33B to $43B per year in the United States. Other health economists have included direct measures of income lost to families of children with ADHD, such as time parents spend away from work (Kvist, Nielsen, & Simonsen, 2013), increased child-care expenses, work loss and stress-related illnesses (Pelham, Foster, & Robb, 2007). Carers of children with ADHD, most often women, report that whilst supporting their children they have limited capacity to obtain high-paid employment (Litt, 2004). In all these studies, having a child with ADHD is framed as cause of low SES as it limits ability to find work and sustain social networks, leading to lost income and social exclusion.

Pathways 6 and 7 (Figure1) illustrate association due to labelling bias**.** Here, clinicians would be more likely to diagnose ADHD in low SES groups (pathway 6, Figure1). In pathway 7, families with socioeconomic disadvantages would be more likely to report ADHD symptoms, perhaps prompted by teachers or difficulties at school.

Where possible, we aimed to assess evidence for some examples of potential pathways in Figure1 through secondary analysis of data from the Millennium Cohort Study (MCS). Because of the longitudinal nature of the study, it was possible to seek evidence for the reverse causality model (Pathway 5) by assessing the effect of having a child with ADHD on socioeconomic factors over 7 years. The questions tested were:-

1. Are parental relationships more likely to dissolve after childhood ADHD has been identified?
2. Does family income decrease for families with a child with ADHD relative to those with a child without?

It was also possible to check for labelling effects through comparison of parent-reported symptom levels with identification by health professional/diagnosis (Pathways 6/7):

1. Do doctors and health professionals diagnose ADHD more often in socioeconomically deprived groups, compared to parent and teacher reports of ADHD symptoms?

Finally, two risk factors consistently implicated in the literature were tested for mediating effects. These were smoking in pregnancy (an early environmental exposure representing Pathway 1) and lack of parent attachment/family conflict (an example of later ‘exposure’ or differential family context, representing Pathway 2). The questions raised were

1. Does low SES mean that mothers are more likely to smoke in pregnancy, leading to greater rates of ADHD?
2. Does low SES affect parenting adversely, increasing the odds of a child having ADHD?

These exposures were intended to be illustrative examples of plausibility of mediation by differential exposure to risk factors as a pathway from socioeconomic disadvantage to ADHD.

## Methods

### Sample

The MCS has followed 19,519 UK children, born between 2000 and 2002, via surveys and direct cognitive testing, carried out by trained interviewers face-to-face in family homes. Information was gathered from the first MCS survey when children were 9 months old, and three, five and 7 years of age: four sweeps of data collection. Informed parental consent was obtained at each stage of the study; the MCS ethical review gives details (Shepherd, 2012). Sample design in MCS was geographically clustered and disproportionately stratified to oversample children from ethnic minorities, and disadvantaged neighbourhoods (details of sample design are in Hansen, 2012). Attrition is a problem common to all longitudinal cohorts and oversampling was used to ensure adequate representation of the population at later ages (Plewis, 2007). Standardised weightings were applied to make the data representative of the UK population, and these adjusted results for the effects of attrition by age 7: approximately 72% of participating families were responding by this stage. We excluded children who had a statement of special needs (*n* = 318) as a proxy for other disorders (i.e. children with autism, hearing problems, conduct disorder were likely to have statements) because being diagnosed with alternative problems could confound the relationship between ADHD and SES (as symptoms of other disorders often co-occur with hyperactivity and some are linked to SES). Children who were twin or triplet siblings were also omitted as the study was underpowered to examine within-family variance. At sweep 4, the mean age was 7.2 years (*SD* = 0.2; age range = 6.3–8.2). The included sample size in this study who had reported on their child's ADHD status was 13305.

Details of fieldwork, coding and questionnaires for MCS measures used are documented at length by Hansen (2012). Extensive documentation and all questionnaires used to generate MCS data are freely available, together with the dataset itself, and can be accessed via the MCS website. The MCS is an ongoing resource, and data collected at further sweeps will be released regularly as children mature.

### Measure of ADHD diagnosis

Parent-reports of ADHD diagnosis by a medical doctor or health professional were taken as ADHD case definition (*n* = 187). This measure has been used to estimate the prevalence of ADHD (Akinbami et al., 2011; Pastor & Rueben, 2008) using US data from National Health Interview Survey (NHIS). The MCS used an adapted version of the NHIS question to record ADHD status: during face-to-face interviews, parents or carers were asked:

1. Has a doctor or health professional ever told you that (sample child) had attention deficit hyperactivity disorder (ADHD)?

In line with other studies (e.g. Boyle et al., 2011) a positive answer to the above question was taken as representative of ADHD diagnosis. Families who answered ‘don't know’ or refused to answer were excluded from the analysis. In MCS, after weighting, 1.5% of children were reported as having been identified/diagnosed with ADHD by sweep 4 in MCS (Russell, Rodgers, Ukoumunne & Ford, 2013).

### Measures of SES

Measures of socioeconomic status taken at all sweeps included parents' highest educational qualification, social class (NS-SEC seven class structure; Office for National Statistics, ONS, 2013), family size and type of housing tenure: in the United Kingdom, social housing is let at low rents and on a secure basis to people in housing need. Equivalised family income was measured at each sweep (adjusted for the number of children per family), with households classed as living in poverty at sweep 4 if their income was equal to or less than 60% of the median household income for the United Kingdom, the definition of poverty set by the UK government (below £236 per week). Family structure (either lone parent or couple) was reported at each sweep. Married couples were more economically advantaged in MCS than lone parent families (Kiernan & Mensah, 2009). The first MCS survey recorded the children's birth weight from the UK Birth Registration and Maternity Hospital Episode Data and the age of mother at childbirth. An ‘index of SES’ was also created from variables measured at sweep 1 that were relatively stable over time: fathers' social class, mothers' social class and paternal and maternal education. To test tobacco use in utero as a mediator, it was necessary to assume the SES index would have preceded pregnancy 18 months previously (assumption of stability; Cole & Maxwell, 2003). The index of SES was calculated by taking the mean values of these measures using an incremental score of 1 for each decrease in rank. If data were missing, the mean across the number of variables for which valid data were recorded was taken. As a check, we generated a second SES index from factor analysis of the same measures (one factor resulted). Correlation between these two indices of SES was 99.5%.

### Risk factors

Records of whether mothers smoked during pregnancy were taken when children were aged 9 months old. Pregnant mothers were classified as smokers or non-smokers. Missing data were not analysed. The Child–Parent Relationship Scale (CPRS) adapted from the Student–Teacher Relationship Scale (Pianta, 1995) was used to measure attachment. The CPRS is a 15 item self-administered rating scale, with responses on a 5-point Likert scale. Items were derived from attachment theory and the attachment Q-set (Waters & Dean, 1985). The items involve the respondent's feelings and beliefs about the relationship with the child, and about the child's behaviour towards the parent. CPRS was measured in MCS sweep 2 (mean age children = 3.1 years, *SD* = 0.2) and used to generate ‘Conflict’ and ‘Closeness’ scores. The ‘closeness’ score was reversed and scores were combined to create a family conflict/distant parent score. Approximately 98% of respondents were mothers.

### Symptoms of ADHD

The Strengths and Difficulties Questionnaire (SDQ) is a behavioural screening questionnaire for children aged 4–16 (Goodman, 2001) that includes a subscale for hyperactivity-inattention and the accompanying impact of problematic behaviours. The SDQ was administered to both parents and teachers at sweep 4 in MCS. The four measures of SDQ teacher/parent-reported hyperactivity-inattention and impact for each child were considered as an indicative of ADHD symptoms as they have been strongly correlated with ADHD in several other studies (e.g. Ullebo, Posserud, Heiervang, Gillberg, & Obel, 2011). Children's clinicians were not informed of SDQ research ratings.

### Analysis

First, the association between the outcome of ADHD diagnosis and a range of indicators of socioeconomic disadvantage, including maternal education level, poverty, income, lone parenthood, family size, birth weight, being a younger mother, and index of SES was established using logistic regression. Standardised weights accounted for attrition and disproportionate sampling in MCS. The odds ratios (OR) from the analyses indicate the increase in odds of being identified with ADHD corresponding to an incremental increase in each predictor.

The reverse causality hypothesis (Pathway 5, Figure1) was that having a child with ADHD causes greater socioeconomic disadvantage and therefore predicts less increase in income for families of children with ADHD and more family breakdown. This was modelled using change in income and family structure. Linear regression was used to compare the increase in income between families whose study child had diagnosed ADHD and families whose child did not have ADHD or a statement of SEN. All cases where family income was recorded at all four time points (when the study child was aged 9 months, 3 years, 5 years and 7 years) were included (*n* = 8193). Further sensitivity analysis utilised propensity score matching to define a control group who had comparable socioeconomic disadvantages to families with a child with ADHD when their children were 9 months old. Nearest neighbour matching was used to define controls, with income at 9 months, child's sex, mother's highest qualification, and lone parenthood as conditioning variables. Study children in control families had neither diagnosis of ADHD, nor statement of SEN by age 7. Linear regression was again used to compare the increase in family income between cases and controls over the 7-year study period. In addition, change in the number of single parent families was plotted for children with ADHD over time.

To test for labelling effects, the association between the outcome of ADHD diagnosis and measures of SES was again modelled using logistic regression, but adjusting for the effects of parent/teacher-reported hyperactivity-inattention symptoms and their impact (*n* ranged from 7826 to 8015: only cases with complete data for all values were included). This was to establish whether the association between clinical ADHD diagnosis and socioeconomic disadvantage existed independently of levels of parent–teacher reported symptoms, i.e. a clinical labelling bias.

Two risk factors that have been repeatedly identified in the literature, smoking in pregnancy, and family conflict/distant parenting, were tested for mediating effects. These were hypothesised to mediate the association between ADHD and SES. For mediation to be inferred, the predictor must precede the mediator, which in turn must precede the outcome, in this case, SES first, mediator second and ADHD third (Cole & Maxwell, 2003). The mediation analysis computed total effects (overall association between index of SES and ADHD with no mediation), indirect effect (for SES-ADHD pathway mediated by measure of interest) and direct effect sizes (for pathway in mediation model not flowing through measure of interest) calculated from a product of coefficients, using bootstrapping (300 replications) to estimate bias corrected confidence intervals (CIs), as recommended by Preacher and Hayes (2008). Figure 3 (in results section) illustrates the causal pathways tested. The indirect effects and direct effect add up to the total effect. The coefficients were standardised to compare direct and indirect effects. The Stata command for binary mediation (Ender, 2011) was used to calculate the indirect effects. This employs a combination of linear regression with logit models. Where the CIs from the indirect effects (the effect of the predictor on the outcome via the mediator) do not cross zero, the analysis provided evidence that mediation had occurred.

## Results

ADHD was strongly associated with a range of indicators of social and economic disadvantage in this cohort, including poverty, housing tenure, income, lone parenthood, index of SES and being a younger mother. Table1 shows descriptive statistics for families who have cohort children with and without a diagnosis of ADHD.

**Table 1:** The association of indicators of socioeconomic disadvantage with ADHD in the Millennium Cohort

Socio-demographic factors	Mean/%		Unadjusted	
	ADHD	No ADHD^a^	OR (95% CI)^b^	*p*
Birth weight (kg)	3.32	3.37	0.86 (0.62,1.19)	0.369
Maternal (years) age at childbirth	26.22	28.45	0.94 (0.91, 0.97)	<0.001
Family income (£ per week)– sweep 4^c^	324	391	0.23 (0.94, 0.55)	0.001
Family size: overall – sweep 4^d^				
Only child	19	13	Referent	0.121
1 sibling	38	46	0.55 (0.34, 0.90)	
2 siblings	27	27	0.68 (0.40, 1.14)	
More than 2 siblings	16	14	0.78 (0.45, 1.34)	
Maternal education: overall				
No qualifications	28	17	Referent	<0.001
School level	59	58	0.61 (0.40, 0.93)	
Degree or higher	13	26	0.32 (0.18, 0.55)	
Poverty				
Above poverty line – sweep 4	60	71	Referent	0.009
Below poverty line	40	29	1.65 (1.13, 2.41)	
Family structure				
Two parent family – sweep 4	63	78	Referent	<0.001
Single parent family	37	22	2.07 (1.42, 3.03)	
Housing tenure – sweep 4: overall				
Social housing,%	44	25	Referent	<0.001
Rent private,%	14	10	0.80 (0.45,1.41)	
Home owner,%	42	65	0.37 (0.26,0.53)	
Index of SES – sweep 1 (higher score = lower SES)	5.02	4.41	1.29 (1.15, 1.45)	<0.001

a includes all children without diagnosis of ADHD, and without Statement of Special Educational Needs.

b number of observations ranges from 11655 to 13305, scores not standardised therefore Odds Ratios (OR) not directly comparable.

c OR shows decreased chances of having ADHD per £1000 increase in weekly income.

d For all categorical variables, as the odds of being in the reference category are 1.

As Table1 illustrates, a larger proportion of children with ADHD diagnosis came from families below the poverty line than in the UK population as a whole. The mean equivalised income for households with an ADHD study child was £324 per week as opposed to £391 for families without a child with ADHD diagnosis, and the odds of parents who owned their own houses having children with ADHD were roughly a third the odds for those who were in social housing. The mean age of mothers at delivery was 26 years for children who would later have ADHD diagnosis and 28 years for the rest of the population. The odds of having a child with ADHD were higher for younger mothers. Mothers with no qualifications were more than twice as likely to have children with ADHD than those with degrees. Lone parents were more likely to have children with ADHD diagnosis than those families with two live-in parents. Greater socioeconomic disadvantage as measured by the index of SES was also associated with ADHD. There was no association between ADHD and birth weight or family size in MCS.

### Checking for reverse causality

**Figure 2:**
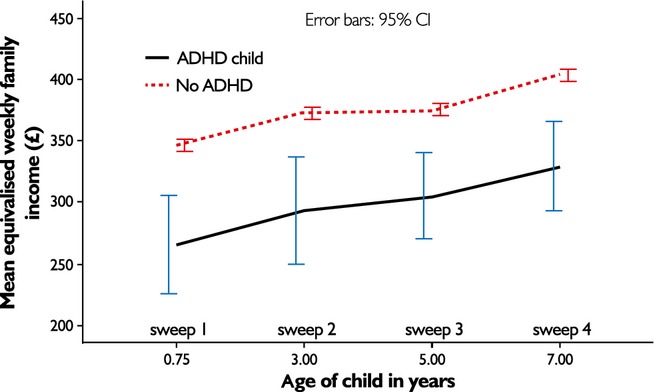
Comparison of mean equivalised income over time in MCS families with study child who had diagnosis of ADHD (*n* = 187) and families whose study child had no ADHD diagnosis (*n* = 13,000+)

Change in income over time for families with and without a study child with ADHD was plotted (Figure2). Overall, income showed a linear trend to increase over time, with income increasing on average £13.93 per year per family who had a child with ADHD (95% CI 8.66–19.19; *p* < .001), and £10.99 for the rest of the sample (95% CI 9.92–12.06; *p* < .001). As Figure2 illustrates, there was no evidence of a comparative decrease in income over time for families with a child with ADHD compared to those without. In fact, there was a slight increase in income for families with a child with ADHD, compared to the rest of the population over the 7-year period, but the difference in income increase was not significant. Results provided no evidence to support the reverse causality model in relation to loss of income

After propensity score matching, change in income with time was also not significantly different between the two groups. Control families' initial average weekly income was £244 as opposed to £249 for families with a child with ADHD. Over the 7-year study period, income increased by £11.63 per year (95% CI, 7.13–16.13) for controls. Families with a child with ADHD child did slightly better, as noted above, although confidence intervals overlapped substantially.

According to the reverse causality hypothesis, a child with ADHD might put additional strain on family resources leading to increase in marital breakup. ADHD is rarely identified before age 3; hyperactive and inattentive behaviours are highly prevalent, and considered ‘normal’ in many toddlers, not just those who go on to a diagnosis of ADHD (Einarsdottir, 2008). We therefore hypothesised an increase in marital breakdown in families after age 3 during primary school years, when ADHD behaviours become problematic. In MCS, there was no discernible increase in percentage of lone parent families in the ADHD group after the age of 3. There was an association between lone parenthood and childhood ADHD, but this was true at 9 months and at 3 years, before ADHD behaviours typically become challenging.

### Checking for clinical labelling bias

Logistic regression reported in Table1 was repeated, but adjusted for parent and teacher-rated symptoms of ADHD. The remaining association between ADHD and SES is reported in Table2. No association of clinical diagnosis with social disadvantage remained independently of parent–teacher-rated symptom level: adjustment accounted for every significant association between ADHD diagnosis with measures of SES. Results suggest that socioeconomic labelling practices do not differ substantially between doctor's diagnosis of ADHD and parent–teacher ratings of symptoms of ADHD.

**Table 2:** The association of indicators of socioeconomic disadvantage with ADHD adjusted for parent and teacher SDQ hyperactivity & impact subscales

Sociodemographic factors	OR (95% CI) adjusted	*p*
Birth weight (kg)	0.96 (0.59, 1.57)	0.891
Maternal (years) age at childbirth	0.99 (0.95, 1.03)	0.721
Family income (£1000)– sweep 4	0.92 (0.26, 3.19)	0.892
Family size: overall – sweep 4		
Only child	Referent	
1 sibling	1.04 (0.44, 2.46)	0.924
2 siblings	1.36 (0.64, 2.87)	0.409
More than 2 siblings	0.85(0.32, 2.27)	0.752
Maternal education: overall		
No qualifications	Referent	
School level	0.69(0.35, 1.34)	0.275
Degree or higher	1.17(0.55, 2.46)	0.680
Poverty		
Above poverty line – sweep 4	Referent	
Below poverty line	1.07 (0.62, 1.86)	0.803
Family type: –sweep 4		
Dual parent	Referent	
Single parent family	1.11(0.61,2.03)	0.734
Housing tenure – sweep 4: overall		
Social housing,%	Referent	
Rent private,%	1.12 (0.44, 2.86)	0.806
Home owner,%	0.94 (0.55, 1.62)	0.829
Index of SES: – sweep 1	1.03 (0.86, 1.24)	0.648

### Checking for mediation

Both smoking during pregnancy and family conflict/distant parenting were independently associated with all the measures of SES. Where more conflict and less closeness was reported between parent and child, families were more likely to experience social or economic disadvantage. Distant parenting/family conflict was also associated with having a child with ADHD, OR = 1.11, 95% CI (1.08–1.13), *p* < 0.001; even after adjustment for measures of socioeconomic disadvantage OR = 1.09, 95% CI (1.05–1.21), *p* < 0.001.

In MCS, 5239 mothers reduced their tobacco use during pregnancy with 2327 mothers giving up smoking, and 1664 continuing to smoke: these were more likely to be from low SES backgrounds. Mothers were more likely to have a child with ADHD if they smoked during pregnancy: OR = 2.26, 95% CI (1.54–3.31) *p* < 001. Smoking in pregnancy was still independently associated with ADHD after adjusting for salient measures of SES, although its effect was weaker, OR = 1.53, 95% CI (1.03–2.29) *p* = 0.036.

**Figure 3:**
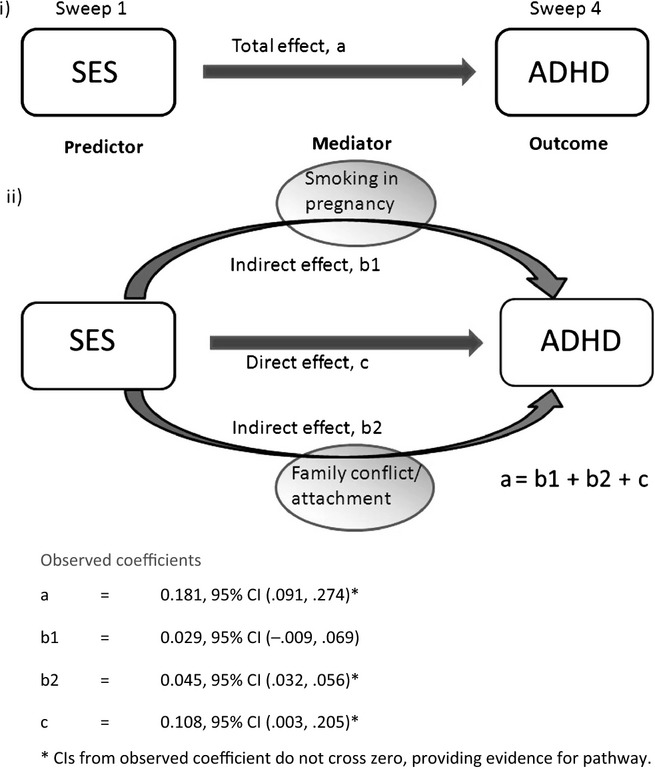
Causal pathway with effect sizes for mediated and nonmediated pathways from SES to ADHD: (i) nomediator: (ii) two mediators

Figure3 shows the effect sizes for mediated and nonmediated pathways from SES to ADHD. The CIs of the indirect effect through parenting do not cross zero, which suggests that parenting may act as a mediator between SES and ADHD. This is a necessary condition for claiming the predictor, mediator and outcome variables are causally related. In contrast, the model does not support smoking in pregnancy as a potential mediator.

## Discussion

This study detected a higher prevalence of ADHD among socioeconomically disadvantaged groups, a finding that concurs with results from a wide range of other studies (Akinbami et al., 2011; Döpfner et al., 2008; Ford et al., 2007; Froehlich et al., 2007; Pastor & Rueben, 2008; Sciberras et al., 2011; St Sauver et al., 2004). To our knowledge, the only systematic review that has touched on this subject was focused on treatment of ADHD and not symptoms or diagnosis of ADHD *per se* (Charach et al., 2011). A recent systematic review of child mental health more generally found socioeconomically disadvantaged individuals were two to three times more likely to develop mental health problems (Reiss, 2013).

Models from health economics have conceptualised ADHD as a disorder with socioeconomic consequences for families (Doshi et al., 2012; Litt, 2004). This study found no evidence for such a reverse causality hypothesis. Kvist et al. (2013) analysed labour supply (i.e. number of days taken off work by mother or father per year) and found parents of children with ADHD took 2–4 additional days off work compared to controls. The UK MCS data did not report on number of days absent from work. Even if it differed for the parents of children with ADHD, it did not impact growth in family income for these families over the 7 years studied, which is considered a more influential measure of SES than labour supply.

We found no support for the hypothesis of overreporting by clinicians about children of lower SES: while clinical diagnosis of ADHD was elevated in socioeconomically disadvantaged groups, our results suggest that parent-rated and teacher-rated symptoms were equally elevated in disadvantaged groups. That is, pathway 6 did not appear greater than pathway 7 in our schematic model (Figure1). It is possible that all parties overreport and overdiagnose ADHD in disadvantaged groups, but as socioeconomic disadvantage subsequently seems to act as a barrier to treatment after clinical diagnosis of ADHD is made (Froehlich et al., 2007), this seems unlikely.

Tobacco use in pregnancy is a suspected risk factor for ADHD (Linnet et al., 2003) and there is a strong relationship between low SES and tobacco use during pregnancy, but there was no evidence to suggest mediation in our analysis. Our results suggest lack of parent involvement/family conflict may be mediating the influence of SES on the outcome of ADHD, and lack of parental involvement/family conflict is more common among families of low SES both in our data and elsewhere (Aber, Bennett, Conley, & Li, 1997). With this mediator added to the equation, the relationship between the measures of SES and ADHD was partially accounted for, but not totally explained.

Parental conflict/attachment in early childhood operated as a mediator, which suggests that family context/'exposures' continue to have an influence throughout the life course. Life course models do not necessarily rule out critical periods of development. Children may be more susceptible at some stages of development to certain risk factors, but differential effects may continue as children mature, and be mitigated by better circumstances later.

The focus of this study is not mediator-specific, but attempts to examine plausibility of mediation and other competing pathways as explanations. The developing child is influenced by an interconnected set of environmental influences and contexts, some related to SES, such as nutrition, disease, sociocultural values, poverty, parenting and peer influences: each may influence outcomes more or less at different developmental stages.

Our study has a number of strengths. The large sample size and longitudinal nature of the dataset has allowed us to infer causal direction by tracking over time. Furthermore, the measures used are well established and MCS has recorded detailed socioeconomic indicators. The greatest limitation to the design was that we were not able to account for genetic predisposition and its potential confounding effect. It was not possible to weight the data in analysis of mediation; however, unweighted regression models are often robust in large datasets (see Wolke et al., 2009). Although the analysis explored parental attachment/family conflict as a simple mediating factor, parenting itself may be influenced by shared genetic predisposition, as well as the effect of having a hyperactive child, hence the bidirectional nature of the arrow in Figure1, pathway 2. There is evidence to suggest treatment with Methylphenidate improves family functioning, for example (Barkley, Karlsson, Pollard, & Murphy, 1985). The influence and character of parenting is likely more complex than acting as a simple mediating factor.

Overall, results provided no evidence for the reverse causality model, or of labelling bias: instead, findings suggest that mediators linked to SES or genetic confounds may provide the most useful framework to explain why ADHD occurs more often in socioeconomically disadvantaged groups (Pathways 1–4, Figure1). The aetiology of ADHD is likely to be a complex interplay of genetic and environmental factors, some linked to socioeconomic disadvantage. Bronfenbrenner (1979) posits a contextual systems model of child development that considers proximal and distal factors that affect how individuals with innate differences react to given environments. As the association between childhood ADHD and socioeconomic disadvantage appears increasingly robust, it becomes important to search for possible explanations for the link. Meta-analysis across many studies is required to substantiate the extent of the association across cultures. Our findings need to be replicated in other datasets, at other developmental stages, and indicate the need for research to examine further potential pathways, especially controlling for genetic predisposition.

##

**Key points**

- Childhood attention deficit hyperactivity disorder (ADHD) and its behavioural symptoms have often been associated with socioeconomic disadvantage.
- In a 2008 sample representative of the United Kingdom, ADHD was associated with a range of indicators of social and economic disadvantage.
- The study provided no evidence to suggest childhood ADHD was a causal factor of socioeconomic disadvantage and no evidence of labelling bias.
- Parent attachment/family conflict apparently mediated the relationship between ADHD and SES.
